# Acute Effects of Anthocyanin-Rich Blackcurrant Extract on Individual Cardiovascular and Metabolic Responses During Supine Rest and Moderate-Intensity Walking in Healthy Men

**DOI:** 10.3390/nu18101631

**Published:** 2026-05-21

**Authors:** Mark E. T. Willems, Pelin Bilgiç, Stefano Montanari, Mehmet A. Şahin

**Affiliations:** 1 School of Sport, Science and Engineering, University of Chichester, Chichester PO19 6PE, UK; stefano.montanari@northampton.ac.uk (S.M.); akifmehmetsahin@gmail.com (M.A.Ş.); 2 Department of Nutrition and Dietetics, Hacettepe University, Ankara 06100, Turkey; pbilgic@gmail.com; 3 Faculty of Arts, Science and Technology, University of Northampton, Northampton NN1 5PH, UK

**Keywords:** anthocyanins, acute effects, dosing, cardiovascular, rest, exercise

## Abstract

**Background/Objectives**: Chronic intake for 7 to 14 days of anthocyanin-rich blackcurrant extract or powder has been shown to alter cardiorespiratory and metabolic responses during rest and moderate-intensity exercise. Whether the observed effects were due to the final intake on the day of testing is not known. We examined whether there were effects of an acute intake of blackcurrant extract on the cardiorespiratory and metabolic responses during supine rest and moderate-intensity treadmill walking. **Methods**: Healthy men (*n* = 15, age: 24 ± 6 years, body mass index: 24.4 ± 4.5 kg·m^−2^) volunteered in a randomized, cross-over designed exploratory study. Acute intake effects of blackcurrant extract (210 mg of anthocyanins) were compared to a control condition. Hemodynamic recordings and indirect calorimetry techniques were used to record physiological and metabolic responses during 10 min of supine rest and 30 min of moderate-intensity treadmill walking. **Results**: At rest, there may have been an effect for an increase in cardiac output (*p* = 0.088). Based on the smallest worthwhile change (i.e., 0.2 × the standard deviation in the control condition), eight participants were classified as responders with an increase in cardiac output of 13.5 ± 8.4% (range: 4.0 to 24.7%). For total peripheral resistance, a decrease was observed (*p* = 0.048, d = −0.40, small effect size), with nine participants classified as responders with a decrease of 17.5 ± 6.1% (range: −9.7 to −28.0%). No changes were observed for other cardiorespiratory and metabolic parameters during supine rest. During moderate-intensity exercise, only heart rate was lower by 2 beats·min^−1^ for the cohort (d = −0.11, trivial effect size) with four participants considered responders when presenting a heart rate lower than the smallest worthwhile change of 3 beats·min^−1^. **Conclusions**: An acute intake of a blackcurrant extract with 210 mg of anthocyanins may have had an effect on vascular regulation mechanisms affecting total peripheral resistance and cardiac output during rest in at least ~50% of the male participants. No acute effects were observed for metabolic responses during rest and exercise. Our findings regarding the metabolic effects are not consistent with previous chronic dosing studies suggesting that repeated daily intake with a dose of 210 mg of anthocyanins is needed to alter substrate oxidation at rest and during moderate-intensity exercise.

## 1. Introduction

Anthocyanins are flavonoid polyphenols [1] with presence in plant-based foods and products [2]. Foremost, dark-colored fruits such as blackcurrant, blackberry, mulberry, black raspberry, maqui berry and blueberry are anthocyanin-rich, i.e., anthocyanins constitute more than 50% of the polyphenol content, with each having a unique anthocyanin profile [3]. Blackberry, for example, contains primarily cyanidins (93%), blackcurrant delphinidins (58%) and cyanidins (42%), and blueberry malvidins (34%), petunidins (23%) and delphinidins (29%) [3]. The regular consumption of anthocyanins in habitual diets is known to provide meaningful health benefits, linked with the potential of anthocyanins and anthocyanin-induced metabolites to provide antioxidant and anti-inflammatory effects (for reviews, see [4,5,6]). In addition, the physiological consequences of the effects of anthocyanin intake on vasodilatory properties [7] and thus on blood flow [8,9] by affecting endothelial function can have applications for exercise performance and recovery for athletes and recreationally active individuals. Among the dark-colored fruits, it is particularly worth noting the numerous studies with chronic intake of anthocyanin-rich blackcurrant that provided meaningful observations (for reviews, see [10,11,12]). However, studies on the effects of anthocyanins have not always provided consistent observations, possibly due to the use of different dosing strategies and exercise modalities. In addition, habitual diet, training status, ethnicity and age may also need to be considered as contributing factors to the inconsistency of observations.

An acute dose of 315 mg of blackcurrant anthocyanins enhanced 5 km treadmill running performance by 2.8% in trained male runners (VO_2max_: 55.4 ± 6.1 mL·kg^−1^·min^−1^) but without an effect on cardiorespiratory and metabolic responses (e.g., heart rate, minute ventilation and fat oxidation) [13]. In addition, an acute dose of 4.3 mg of blackcurrant anthocyanins per kg body mass in trained female and male cyclists also did not alter the cardiorespiratory responses during a maximal incremental cycling test and a 4 km cycling time trial and did not improve the time-trial time [14]. However, the absence of cardiorespiratory and metabolic responses in the works of Moss et al. [13] and Morton et al. [14] may be due to the nature of performing exercise tasks with high intensity, but this is speculative. In contrast, Montanari et al. [15] showed, in a home-based study, that an acute intake of blackcurrant extract (i.e., 315 mg of blackcurrant anthocyanins) enhanced 16.1. km cycling performance in slower cyclists but no physiological responses were recorded. Acute dosing studies result in the bioavailability of anthocyanins and anthocyanin-derived metabolites in the blood, whereas in chronic dosing studies, there could be an accumulation, with longer presence of those metabolites affecting cell function.

Chronic dosing studies that included observations during rest and moderate-intensity exercise generally produced more consistent outcomes. Chronic dosing for 7 days with powder (daily 138.6 mg of anthocyanins and 49 mg of vitamin C) made from New Zealand-grown blackcurrant enhanced cardiac output by 26% and lowered total peripheral resistance by 16% during rest in a cohort of male (*n* = 8) and female (*n* = 5) endurance triathletes, but with no effect on cycling-induced cardiovascular responses [16]. Blackcurrant-induced cardiovascular observations were also obtained with chronic dosing of blackcurrant extract. In a dose–response study by Cook et al. [17], cardiac output, for example, was increased by 28% and total peripheral resistance decreased by 20% with 14-day daily intake of 320 mg of blackcurrant anthocyanins. In addition, 7-day intake of blackcurrant extract (daily 210 mg of anthocyanins) enhanced post-exercise hypotension after 1 h of treadmill walking at 50% $\dot{V}$O_2max_ in recreationally active normotensive females and males [18,19]. Blueberry intake for 8 weeks (daily 22 g freeze-dried powder with 469.48 mg of anthocyanins) reduced blood pressure in postmenopausal women with pre- and stage 1 hypertension [20]. However, in chronic dosing studies with blackcurrant anthocyanins to examine cardiovascular and metabolic responses, it cannot be excluded that there can be an effect by the final intake of anthocyanins, sometimes on the day of testing (e.g., [17]).

Therefore, the aim of the present exploratory study was to examine the effects of an acute intake of 210 mg of capsulated blackcurrant anthocyanins on the cardiorespiratory and metabolic responses during supine rest and moderate-intensity treadmill walking. The dose of 210 mg of blackcurrant anthocyanins has been shown to provide beneficial cardiovascular and metabolic effects with chronic dosing, especially during moderate-intensity exercise [21,22,23].

## 2. Materials and Methods

### 2.1. Participants

Healthy recreationally active men (*n* = 15, age: 24 ± 6 years, body mass: 78 ± 16 kg, height: 178 ± 6 cm, BMI: 24.4 ± 4.5 kg·m^−2^, body fat: 15.2 ± 5.1%) volunteered for this study. Following a detailed explanation of the procedures, the participants provided written informed consent. The laboratory visits for the participants were part of a larger study with the overall objective of examining the effect of different dosing strategies with anthocyanin-rich blackcurrant on the cardiovascular, metabolic and physiological responses during supine rest and moderate-intensity walking exercise [21,22,23,24]. All participants were non-smokers and did not have a known allergy to anthocyanin-containing foods and drinks, including berries and berry products. No other dietary supplementation and medicine were allowed during this study. This exploratory study employed a randomized, cross-over experimental design with a control condition. Ethical approval for this study was obtained from the University of Chichester Research Ethics Committee (ethical approval code: 1718_34), with the protocols and procedures conforming to the 2013 Declaration of Helsinki. For the present study on the acute effects of anthocyanin-rich blackcurrant extract, the participants had three morning visits to the exercise physiology laboratory.

### 2.2. First Visit and Standardization of Walking Speed at Moderate Intensity

Body mass, height, and body fat % (Tanita BC418 Segmental Body Composition analyzer, Tanita, IL, USA) were measured in the first visit. A food frequency questionnaire on the consumption of anthocyanin-containing foods and drinks listed in the Phenol Explorer database [25] was completed by the participants to estimate daily anthocyanin intake (86 ± 74 mg·day^−1^) (range: 0.1 to 206.8 mg·day^−1^). The short version of the International Physical Activity Questionnaire [26] was completed by the participants to quantify the habitual physical activity level (i.e., 4534 ± 1576 MET·week^−1^). In the first visit, the 1-MET, i.e., the oxygen consumption at rest, was measured with two expired air collections of 10 min each with the participants lying supine on an examination table. During expired air collection, the laboratory air fractions of oxygen and carbon dioxide were measured. These fractions were taken as the inspiratory fractions of oxygen and carbon dioxide for the participant and used for calculation of the respiratory parameters. The expired air was collected in Douglas bags and analyzed for respiratory parameters (Servomex gas analyzer, Series 1400, Crowborough, UK) and volume (Harvard Apparatus Ltd. (Holliston, MA, USA) with a dry gas meter). Douglas bags are the gold standard for collection of expired air [27]. Gas volumes were calculated using the Haldane transformation and standardization to STPD conditions with consideration of the inspiratory fractions for oxygen and carbon dioxide. Oxygen consumption for the 10 min recording with the lowest minute ventilation was taken as the 1-MET value for each participant. For male participants, 1-MET was 3.97 ± 0.66 mL·kg^−1^·min^−1^ (95% CI [3.61, 4.34]). For familiarization purposes, during the 10 min expired air collections at rest, cardiovascular measurements were obtained with a beat-to-beat blood-pressure-monitoring system (Portapres^®^ Model 2, Finapres Medical Systems BV, Amsterdam, The Netherlands) using the arterial volume clamp method [28]. For determination of the walking speed that provided moderate-intensity exercise, the participants performed an incremental walking test on a treadmill (HP Cosmos Pulsar Bodycare Products, Southam, UK) with walking speeds of 2, 3, 4, 5, and 6 km·h^−1^, each speed for 8 min with a 2 min break between each speed. In the final 3 min at each speed, expired air was collected in Douglas bags. The linear relationship between walking speed and oxygen uptake allowed for the calculation of the participant’s moderate-intensity walking speeds.

### 2.3. Second and Third Experimental Visits

For the acute-intake condition, participants consumed two capsules of NZBC extract (600 mg containing 210 mg of anthocyanins, i.e., 35–50% delphinidin-3-O-rutinoside, 5–20% delphinidin-3-O-glucoside, 30–45% cyanidin-3-O-rutinoside, 3–10% cyanidin-3-O-glucoside) (CurraNZ, Health Currancy Ltd., Surrey, UK) two hours before arriving at the laboratory. Czank et al. [29] observed maximum plasma anthocyanin (i.e., cyanidin-3-O-glucoside) concentration and the presence of anthocyanin metabolites 2 h following intake of cyanidin-3-O-glucoside. Participants were instructed to have one slice of bread and water 3 h before the visits. In addition, the participants completed a 48 h food diary before both visits and were advised to match the 48 h dietary intake for each of the visits. Food diaries were analyzed using Nutritics (Nutritics Ltd., Dublin, Ireland) for carbohydrate, fat, protein, and total energy intake. There were no differences in absolute values for carbohydrate, fat, protein, or total energy intake between the control and blackcurrant conditions (*p* > 0.05) (Table 1). For the control condition, no supplement was consumed. There was at least two weeks between testing conditions. The participants did not report any adverse reactions by the intake of New Zealand blackcurrant extract. For experimental visits two and three, the measurements consisted of the respiratory and cardiovascular responses during supine rest for 2 × 10 min and 30 min of moderate-intensity walking. All measurements were done within two hours following arrival to the laboratory. In the control and acute intake conditions, the cardiovascular responses during the 10 min with the lowest minute ventilation were taken as resting cardiovascular responses. The moderate-intensity walking speed for three participants was decided to be 4-MET because those participants indicated that the treadmill speed at 5-MET would require jogging. Walking speed for moderate-intensity exercise for the two experimental visits was 5.7 ± 0.7 km·h^−1^ (95% CI [5.3, 6.1]).

### 2.4. Data and Statistical Analysis

Gas volumes were calculated using the Haldane transformation and standardized to STPD conditions with consideration of inspired fractions of oxygen and carbon dioxide recorded in the laboratory during each expired air collection. Respiratory exchange ratio (i.e., RER) was calculated as the ratio between the carbon dioxide produced and oxygen consumed. The rates of whole-body fat and carbohydrate oxidation were calculated with equations from Frayn [30] and the assumption of negligible protein oxidation.

$$Fat\;oxidation\;\left(g\cdot {min}^{-1}\right)=1.67\times \dot{V}O_{2}-1.67\times \dot{V}{CO}_{2}$$

$$Carbohydrate\;oxidation\;\left(g\cdot {min}^{-1}\right)=4.55\times \dot{V}CO_{2}-3.21\times \dot{V}O_{2}$$

In the control and acute-intake conditions, the cardiorespiratory responses during the 10 min of supine rest were analyzed for the 10 min bout with the lowest minute ventilation. During the treadmill walk, the cardiorespiratory responses were measured at 7–10, 17–20 and 27–30 min and averaged. Statistical analyses were completed using Graphpad Prism (GraphPad Prism version 5.00 for Windows, GraphPad Software, San Diego, CA, USA). Normality was tested with the Shapiro–Wilks normality test. Absolute values of the cardiovascular responses for the control and blackcurrant conditions during supine rest and during moderate-intensity exercise were analyzed using Student’s paired *t*-tests. Cohen’s d effect sizes were calculated for parameters in the cohort with significant change or those in which there may have been a change and considered trivial (d < 0.2), small (d = 0.2–0.49), moderate (d = 0.5–0.79) and large (d ≥ 0.8), respectively. The sample size of 15 participants was higher or similar to that of previous studies with observations of effects of NZBC extract on cardiovascular responses [e.g., Willems et al. [13] (*n* = 16); Cook et al. [17] (*n* = 15)]. Significance was accepted at *p* < 0.05, with 0.05 ≥ *p* ≤ 0.1 interpreted according to guidelines by Curran-Everett and Benos [31]. Data for the cohort are reported as mean ± SD. Regarding the metabolic and cardiorespiratory parameters that showed significance for change or a likely change [31], the number of individuals identified as responders were considered based on the smallest worthwhile change (SWC, i.e., 0.2 × SD of the control condition) threshold [32,33]. Data for the responders were also reported as mean ± SD.

## 3. Results

### 3.1. Cardiovascular Responses During Supine Rest in Healthy Males for Control and Blackcurrant Conditions

**Figure 1:**
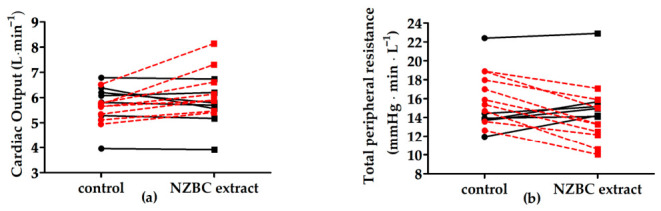
Cardiac output (**a**) and total peripheral resistance (**b**) during supine rest. NZBC, New Zealand blackcurrant. Red data points, responders.

In Table 2 are presented the mean ± SD of the cardiovascular responses during supine rest for the control and NZBC extract conditions. During supine rest with acute intake of NZBC extract (210 mg of anthocyanins), there was no effect on heart rate (control: 95%CI [56, 66 beats·min^−1^], range: 42–75 beats·min^−1^); NZBC extract: 95% CI [57, 68 beats·min^−1^], range: 42–79 beats·min^−1^) and stroke volume (control: 95%CI [88, 102 mL], range: 74–130 mL; NZBC extract: 95%CI [89, 107 mL], range: 75–139 mL). There may have been an effect of an increase in cardiac output (control: 95%CI [5.29, 6.07 L·min^−1^], range: 3.95–6.79 L·min^−1^); NZBC extract: 95%CI [5.45, 6.53 L·min^−1^], range: 3.81–8.14 L·min^−1^) with a small effect size (d = 0.35). The 95%CI for the differences in cardiac output was [−0.05, 0.68 L·min^−1^]. Eight participants were classified as responders with an increase in cardiac output of 13.5 ± 8.4% (range: 4.0 to 24.7%) (Figure 1a).

There were no differences in systolic blood pressure (control: 95%CI [130, 145 mmHg], range: 119–158 mmHg); NZBC extract: 95% CI [126, 140 mmHg], range: 109–150 mmHg), diastolic blood pressure (control: 95% CI [66, 74 mmHg], range: 59–87 mmHg); NZBC extract: 95% CI [64, 71 mmHg], range: 57–75 mmHg), and mean arterial pressure (control: 95%CI [83, 91 mmHg], range: 78–106 mmHg; NZBC extract: 95%CI [80, 88 mmHg], range: 73–96 mmHg). For total peripheral resistance, a decrease with moderate effect size (d = −0.40) was observed with acute intake of NZBC extract (control: 95%CI [14.09, 17.25 mmHg·min·L^−1^], range: 11.96–22.41 mmHg·min·L^−1^; NZBC extract: 95%CI [12.77, 16.14 mmHg·min·L^−1^], range: 10.04–22.90 mmHg·min·L^−1^). The 95%CI for the differences in total peripheral resistance was [−2.42, −0.02 mmHg·min·L^−1^]. Nine participants were classified as responders with a decrease of 17.5 ± 6.1% (range: −9.7 to −28.0%) (Figure 1b). In the cohort, seven participants were classified as responders as they experienced both an increase in cardiac output and a decrease in total peripheral resistance. This would suggest that acute intake of NZBC extract with 210 mg of anthocyanins had an effect on vascular regulation mechanisms, at least in ~50% of the male participants.

### 3.2. Physiological and Metabolic Responses During Supine Rest for a 10 Min Period for Control and Blackcurrant Conditions

In Table 3 are presented the mean ± SD of the physiological and metabolic responses during supine rest for the control and NZBC extract conditions. During supine rest with acute intake of NZBC extract (210 mg of anthocyanins), there was no effect on minute ventilation (control: 95%CI [7.29, 8.92 L·min^−1^], range: 6.42–11.42 L·min^−1^); NZBC extract: 95%CI [7.84, 8.54 L·min^−1^], range: 6.41–9.46 L·min^−1^), oxygen consumption in absolute values (control: 95%CI [0.26, 0.33 L·min^−1^], range: 0.22–0.46 L·min^−1^); NZBC extract: 95%CI [0.27, 0.32 L·min^−1^], range: 0.24–0.44 L·min^−1^), oxygen consumption in relative values (control: 95%CI [3.41, 4.18 mL·kg^−1^·min^−1^], range: 2.92–5.68 mL·kg^−1^·min^−1^); NZBC extract: 95%CI [3.84, 4.21 mL·kg^−1^·min^−1^], range: 2.92–5.18 mL·kg^−1^·min^−1^), carbon dioxide production (control: 95%CI [0.22, 0.28 L·min^−1^], range: 0.17–0.36 L·min^−1^); NZBC extract: 95%CI [0.23, 0.27 L·min^−1^], range: 0.20–0.36 L·min^−1^), carbohydrate oxidation (control: 95%CI [0.14, 0.22 g·min^−1^], range: 0.08–0.29 g·min^−1^); NZBC extract: 95%CI [0.15, 0.22 g·min^−1^], range: 0.11–0.33 g·min^−1^), fat oxidation (control: 95%CI [0.062, 0.097 g·min^−1^], range: 0.033–0.094 g·min^−1^); NZBC extract: 95%CI [0.061, 0.093 g·min^−1^], range: 0.028–0.141 g·min^−1^) or respiratory exchange ratio (control: 95%CI [0.814, 0.864], range: 0.774–0.920); NZBC extract: 95%CI [0.822, 0.870], range: 0.790–0.945). An acute intake of 210 mg of blackcurrant anthocyanins does not affect respiratory and whole-body metabolic responses during supine rest in male participants.

### 3.3. Cardiovascular Responses in Healthy Males During 30 Min of Moderate-Intensity Treadmill Walking for Control and Blackcurrant Conditions

In Table 4 are presented the mean ± SD of the cardiovascular responses during 30 min of moderate-intensity treadmill walking for the control and NZBC extract conditions. During moderate-intensity treadmill walking with acute intake of NZBC extract (210 mg of anthocyanins), the heart rate was lower by 2 beats·min^−1^ (d = −0.11, trivial effect size) for the cohort (control: 95%CI [93, 112 beats·min^−1^], range: 70–130 beats·min^−1^); NZBC extract: 95% CI [90, 110 beats·min^−1^], range: 70–130 beats·min^−1^). The 95%CI for the differences in heart rate was [- 5, 0 beats·min^−1^]. However, only four participants were considered responders and had a heart rate lower than the smallest worthwhile change of 3 beats·min^−1^. There was no effect on stroke volume (control: 95%CI [107, 121 mL], range: 97–136 mL; NZBC extract: 95%CI [108, 128 mL], range: 85–149 mL), cardiac output (control: 95%CI [10.56, 12.73 L·min^−1^], range: 6.76–13.85 L·min^−1^); NZBC extract: 95%CI [10.65, 12.91 L·min^−1^], range: 7.74–15.12 L·min^−1^), systolic blood pressure (control: 95%CI [147, 168 mmHg], range: 123–191 mmHg); NZBC extract: 95% CI [147, 163 mmHg], range: 127–182 mmHg), diastolic blood pressure (control: 95% CI [66, 76 mmHg], range: 57–93 mmHg); NZBC extract: 95% CI [62, 73 mmHg], range: 49–86 mmHg), mean arterial pressure (control: 95%CI [87, 99 mmHg], range: 73–113 mmHg; NZBC extract: 95%CI [84, 96 mmHg], range: 70–115 mmHg), or total peripheral resistance (control: 95%CI [7.22, 9.21 mmHg·min·L^−1^], range: 6.25–13.32 mmHg·min·L^−1^; NZBC extract: 95%CI [6.87, 8.89 mmHg·min·L^−1^], range: 5.69–11.45 mmHg·min·L^−1^). This would suggest that the acute intake of NZBC extract with 210 mg of anthocyanins had no effect on vascular regulation mechanisms during moderate-intensity treadmill walking. However, in studies with trivial effects for cohort observations, the individual responses may show a potential beneficial effect for some participants.

### 3.4. Physiological and Metabolic Responses During 30 Min of Moderate-Intensity Treadmill Walking for Control and Blackcurrant Conditions

In Table 5 are presented the mean ± SD of the physiological and metabolic responses during 30 min of moderate-intensity walking for the control and NZBC extract conditions. During 30 min of moderate-intensity treadmill walking with acute intake of NZBC extract (210 mg of anthocyanins), there was no effect on minute ventilation (control: 95%CI [27.45, 33.93 L·min^−1^], range: 22.10–43.80 L·min^−1^); NZBC extract: 95%CI [28.43, 33.99 L·min^−1^], range: 24.00–41.70 L·min^−1^), oxygen consumption in absolute values (control: 95%CI [1.35, 1.69 L·min^−1^], range: 1.17–2.08 L·min^−1^); NZBC extract: 95%CI [1.35, 1.65 L·min^−1^], range: 1.16–1.99 L·min^−1^), oxygen consumption in relative values (control: 95%CI [17.43, 21.78 mL·kg^−1^·min^−1^], range: 13.56–26.64 mL·kg^−1^·min^−1^); NZBC extract: 95%CI [17.27, 21.52 mL·kg^−1^·min^−1^], range: 13.28–25.99 mL·kg^−1^·min^−1^), carbon dioxide production (control: 95%CI [1.14, 1.46 L·min^−1^], range: 0.91–1.77 L·min^−1^); NZBC extract: 95%CI [1.16, 1.45 L·min^−1^], range: 0.99–1.75 L·min^−1^), carbohydrate oxidation (control: 95%CI [0.739, 1.195 g·min^−1^], range: 0.310–1.890 g·min^−1^); NZBC extract: 95%CI [0.865, 1.231 g·min^−1^], range: 0.580–1.710 g·min^−1^), fat oxidation (control: 95%CI [0.299, 0.431 g·min^−1^], range: 0.175–0.595 g·min^−1^); NZBC extract: 95%CI [0.285, 0.359 g·min^−1^], range: 0.224–0.466 g·min^−1^) and respiratory exchange ratio (control: 95%CI [0.825, 0.878], range: 0.765–0.942); NZBC extract: 95%CI [0.851, 0.884], range: 0.810–0.916). An acute intake of 210 mg of blackcurrant anthocyanins did not affect respiratory or whole-body metabolic responses during 30 min of moderate-intensity treadmill walking in male participants.

## 4. Discussion

The present study provided only limited evidence for an acute effect caused by the intake of an anthocyanin-rich supplement with 210 mg of anthocyanins made from New Zealand-grown blackcurrant on some cardiovascular responses (i.e., total peripheral resistance and cardiac output) during supine rest without affecting the metabolic responses. No acute effect of the intake of blackcurrant extract was observed for the cardiorespiratory and metabolic responses during moderate-intensity walking. The lower heart rate of 2 beats·min^−1^ for the cohort could be due to normal daily variability. Justification for the dose in the present exploratory study was based on dose–response studies with chronic intake of blackcurrant extract that showed meaningful effects on cardiovascular responses during rest and metabolic responses during moderate-intensity exercise [17,34]. In these studies, Cook et al. [17,34] dosed male endurance cyclists for 7 days with 105, 210 and 315 mg of blackcurrant anthocyanins (i.e., 300, 600 and 900 mg of capsulated blackcurrant extract). In the work of Cook et al. [17], the total peripheral resistance in the group of male endurance-trained cyclists (*n* = 15) with a 7-day intake of 210 and 315 mg of blackcurrant anthocyanins was lowered by 20% for both dose conditions during supine rest. In chronic dosing studies with stronger responses to the intake of anthocyanin-rich supplementation, the final intake on the day of testing or days before testing could have contributed to the observed effects [17,33,35,36,37,38]. In the present study, with the acute intake of 210 mg of blackcurrant anthocyanins, we observed in our participants (*n* = 15) a decrease of only 7.8% for total peripheral resistance during supine rest. In addition, it needs to be noted that the significant acute effect was due to a response in only 60% of the participants. It seems that chronic dosing with blackcurrant extract is required to optimize effects on cardiovascular function at rest. During moderate-intensity treadmill walking, no acute effects were observed for cardiorespiratory and metabolic responses. No group studies have addressed both cardiovascular function and metabolic responses to an acute intake with a dose higher than 210 mg of blackcurrant anthocyanins. Therefore, future studies are warranted in recreationally active individuals to examine the acute dose–response effects of blackcurrant anthocyanins on cardiorespiratory and metabolic parameters during rest and exercise.

Within hours, the acute intake of anthocyanins results in the bioavailability of anthocyanins and anthocyanin-derived metabolites in the blood [8,29,39,40,41,42]. It needs to be noted that it is common to have high inter- and intra-individual variability in plasma anthocyanins and metabolites [39,41,42]. The variability in plasma anthocyanins and metabolites may be linked with the variability in cardiovascular responses in the present study. In the work of Matsumoto et al. [8], the elevation in anthocyanins in the blood was obtained with an intake of 17 mg per kg body mass of blackcurrant concentrate with 10.83% of anthocyanins. However, the acute dose in the work of Matsumoto et al. [8] was much lower (e.g., 128 mg for a 70 kg individual) than the dosing with blackcurrant anthocyanins in the present study; nevertheless, it enhanced forearm blood flow by 22% after 2 h of intake. The decrease in total peripheral resistance in the present study with the observation that there may have been an increase in cardiac output suggests enhanced systemic blood flow by acute intake of 210 mg of blackcurrant anthocyanins. During supine rest, however, whether there was an effect of the plasma anthocyanins on endothelial cell function that enabled vasodilation is not known, as effects of anthocyanin-induced metabolites cannot be ruled out [43,44,45]. An in vitro study with exposure of rat thoracic aortic rings to the anthocyanin cyanidin-3-glucoside did show a dose response effect on vasorelaxation [46]. However, in vitro studies have a non-changing exposure for a set duration to a constant concentration of anthocyanins and such exposure is different than in vivo studies. In addition, caution is required to generalize from in vitro findings to in vivo application. In in vivo studies, there is presence in the blood with different anthocyanin amounts together with dynamic bioavailability. Dynamic bioavailability also occurs for anthocyanin-induced metabolites. Costello et al. [39] showed the variability in individual responses to an acute intake of 105 mg of blackcurrant anthocyanins for vanillic acid, protocatechuic acid and gallic acid. For some of these anthocyanin-induced metabolites, i.e., protocatechuic acid, for example, there is also evidence of providing vasorelaxation by affecting human endothelial cells [47]. It is considered a key metabolite from the intake of blackcurrant anthocyanins (i.e., cyanidin-3-glucosides [48] and delphinidin and cyanidin rutinosides [40,49]). Future dose–response studies are warranted to address the relationship between the bioavailability of anthocyanins and anthocyanin-derived metabolites and cardiovascular responses.

It needs to be noted that three participants were tested for responses to blackcurrant at 4-MET. Future studies are warranted as well to examine, in the same cohort, the responses to low- (<3-MET), moderate-intensity (3–6 MET) and high-intensity (>6-MET) exercise [50]. In addition, although the present study had some responders to the acute intake of anthocyanin-rich blackcurrant, the small sample size and not knowing the consistency of response [51] warrants further caution regarding our exploratory findings. A limitation of the present study was that it did not have a placebo-controlled condition. However, as far as we know, the measured parameters are not open for potential bias in the testing environment. In addition, the present observations were part of a larger study with different dosing strategies [21,22,23,24]. In those studies, we did not formally assess the potential for order, carry-over or learning effects. It also needs to be noted that the multiple comparisons with paired Student’s *t*-tests may have resulted in a type I error [52] for the change in total peripheral resistance. Thus, all observations in the present study should be taken as exploratory findings. However, the observations of the present study do not result in cohort recommendations regarding the intake of blackcurrant extract.

## 5. Conclusions

In conclusion, the cardiovascular system during supine rest can respond to an acute intake of anthocyanin-rich extract (i.e., 210 mg of anthocyanins) made from New Zealand-grown blackcurrant with lower total peripheral resistance and may be indicative of enhanced blood flow. However, the response rate to an acute intake of 210 mg of blackcurrant anthocyanins was only about 60%. An enhanced blood flow may potentially support recovery and limit post-exercise symptoms, but this warrants further investigation.

## Figures and Tables

**Table 1:** Absolute values for dietary intake of carbohydrate, fat and protein 48 before the control and NZBC extract conditions.

	Control	NZBC Extract
Carbohydrate (g)	428 ± 111	418 ± 105
Fat (g)	172 ± 67	169 ± 69
Protein (g)	253 ± 88	254 ± 86

Values are mean and SD for 15 male participants. There were no differences between control and NZBC extract conditions.

**Table 2:** Cardiovascular responses during 10 min of supine rest in healthy male participants. NZBC, New Zealand blackcurrant; TPR, total peripheral resistance.

	Control	NZBC Extract	*p*-Value
Heart rate (beats·min^−1^)	61 ± 10	62 ± 10	0.358
Stroke volume (mL)	95 ± 13	98 ± 16	0.443
Cardiac output (L·min^−1^)	5.68 ± 0.71	6.00 ± 0.98	0.088
Systolic blood pressure (mmHg)	137 ± 13	133 ± 13	0.181
Diastolic blood pressure (mmHg)	70 ± 7	67 ± 6	0.156
Mean arterial pressure (mmHg)	87 ± 7	84 ± 7	0.169
TPR (mmHg·min·L^−1^)	15.68 ± 2.85	14.45 ± 3.04	0.048

**Table 3:** Physiological and metabolic responses during 10 min of supine rest in healthy male participants. NZBC, New Zealand blackcurrant.

	Control	NZBC Extract	*p*-Value
Minute ventilation (L·min^−1^)	8.11 ± 1.48	8.01 ± 0.96	0.806
Oxygen consumption (L·min^−1^)	0.30 ± 0.06	0.30 ± 0.05	0.900
Oxygen consumption (mL·kg^−1^·min^−1^)	3.79 ± 0.69	3.84 ± 0.66	0.724
Carbon dioxide production (L·min^−1^)	0.25 ± 0.05	0.25 ± 0.04	0.745
Carbohydrate oxidation (g·min^−1^)	0.179 ± 0.069	0.188 ± 0.062	0.672
Fat oxidation (g·min^−1^)	0.079 ± 0.032	0.077 ± 0.028	1.000
Respiratory exchange ratio	0.839 ± 0.046	0.846 ± 0.044	0.651

**Table 4:** Cardiovascular responses during 30 min of moderate-intensity treadmill walking in healthy male participants. NZBC, New Zealand blackcurrant; TPR, total peripheral resistance.

	Control	NZBC Extract	*p*-Value
Heart rate (beats·min^−1^)	102 ± 17	100 ± 18	0.041
Stroke volume (mL)	114 ± 13	118 ± 18	0.373
Cardiac output (L·min^−1^)	11.65 ± 1.96	11.78 ± 2.04	0.750
Systolic blood pressure (mmHg)	158 ± 18	155 ± 15	0.357
Diastolic blood pressure (mmHg)	71 ± 9	68 ± 10	0.226
Mean arterial pressure (mmHg)	93 ± 10	90 ± 11	0.217
TPR (mmHg·min·L^−1^)	8.21 ± 1.79	7.88 ± 1.83	0.487

**Table 5:** Physiological and metabolic responses during 30 min of moderate-intensity treadmill walking in healthy male participants. NZBC, New Zealand blackcurrant.

	Control	NZBC Extract	*p*-Value
Minute ventilation (L·min^−1^)	30.69 ± 5.85	31.21 ± 5.02	0.212
Oxygen consumption (L·min^−1^)	1.52 ± 0.30	1.50 ± 0.27	0.142
Oxygen consumption (mL·kg^−1^·min^−1^)	19.61 ± 3.93	19.40 ± 3.84	0.185
Carbon dioxide production (L·min^−1^)	1.30 ± 0.29	1.30 ± 0.26	0.767
Carbohydrate oxidation (g·min^−1^)	0.967 ± 0.412	1.048 ± 0.331	0.210
Fat oxidation (g·min^−1^)	0.365 ± 0.120	0.322 ± 0.066	0.134
Respiratory exchange ratio	0.852 ± 0.047	0.868 ± 0.030	0.159

## Data Availability

Data are available on reasonable request from the corresponding author.
